# Prevalence of Diabetes and Impaired Fasting Glucose in Chinese Adults, China National Nutrition and Health Survey, 2002

**Published:** 2010-12-15

**Authors:** Shuqian Liu, Wenyu Wang, Xiaoguang Yang, Elisa T. Lee, Jian Zhang, Yuna He, Jianhua Piao, Chonghua Yao, Zhechun Zeng, Barbara V. Howard, Richard R. Fabsitz, Lyle Best

**Affiliations:** College of Public Health, University of Oklahoma, Oklahoma City, Oklahoma. Dr Liu is also affiliated with the Beijing Institute of Heart, Lung and Blood Vessel Diseases, Beijing Anzhen Hospital of the Capital University of Medical Sciences, Beijing, China.; Center for American Indian Health Research, College of Public Health, University of Oklahoma HSC; National Institute of Nutrition and Food Safety, Chinese Center for Disease Control and Prevention, Key Laboratory of Trace Element Nutrition, Ministry of Health; College of Public Health, University of Oklahoma, Oklahoma City, Oklahoma; National Institute for Nutrition and Food Safety, Chinese Center for Disease Control and Prevention, Beijing, China; National Institute for Nutrition and Food Safety, Chinese Center for Disease Control and Prevention, Beijing, China; National Institute for Nutrition and Food Safety, Chinese Center for Disease Control and Prevention, Beijing, China; Beijing Institute of Heart, Lung and Blood Vessel Diseases, Beijing Anzhen Hospital of the Capital University of Medical Sciences, Beijing, China; Beijing Institute of Heart, Lung and Blood Vessel Diseases, Beijing Anzhen Hospital of the Capital University of Medical Sciences, Beijing, China; MedStar Research Institute, Washington, DC; Epidemiology and Biometry Program, National Heart, Lung, and Blood Institute, Bethesda, Maryland; Missouri Breaks Industries Research, Inc, Timber Lake, South Dakota

## Abstract

**Introduction:**

As a result of rapid economic development in China, the lifestyles and dietary habits of its people have been changing, and the rates of obesity, diabetes, and other chronic conditions have increased substantially. We report the prevalence of type 2 diabetes and impaired fasting glucose (IFG) and the association between diabetes and overweight and obesity in Chinese adults. We also compare the results with those from the US National Health and Nutrition Examination Survey, 1999-2002.

**Methods:**

Data were from adults aged 20 years or older who participated in the China National Nutrition and Health Survey, 2002 (n = 47,729). Diabetes and IFG were defined by the American Diabetes Association 2009 criteria. We assessed the prevalence of diabetes, IFG, and overweight and obesity by sex, age, region of residence, and ethnicity.

**Results:**

The prevalence of diabetes and IFG in Chinese adults was 2.7% and 4.9%, respectively. The prevalence of diabetes increased with age and body mass index. Men and women had a similar prevalence of diabetes, but men had a significantly higher prevalence of IFG. The prevalence of diabetes among Chinese who lived in urban areas was 2 to 3 times higher than the prevalence among those who lived in rural areas (3.9% for urban areas and 6.1% for large cities vs 1.9% for rural areas), and the prevalence of IFG was 1.5 to 2 times higher (6.1% and 8.1% vs 4.2%, respectively). The prevalence of diabetes among Chinese women and young (20-39 y) and middle-aged (40-59 y) adults who lived in large cities was similar to the prevalence of diabetes in the US population.

**Conclusion:**

The prevalence of diabetes and IFG was much higher in urban than rural areas, particularly in the large cities of China. Prevention must be emphasized among adults to reduce the future social and economic burden of diabetes in China.

## Introduction

China has undergone rapid social and economic changes in the last 20 years. The lifestyle and dietary habits of its people have also been changing, and the rates of obesity, diabetes, and other chronic conditions have increased dramatically over the past decades (1). Several previous large cross-sectional studies have reported the prevalence of diabetes in China (2-4). However, these studies either did not use a nationwide population-based representative random sample (2,4) or assessed populations limited by age, geographic region, or economic status (5). The International Collaborative Study of Cardiovascular Disease in Asia (InterASIA) (3,5) considered Chinese adults aged 35 to 74 years, emphasized the geographic and economic differences between northern and southern regions (5 provinces in each), and examined differences between urban and rural areas. The China National Nutrition and Health Survey (CNNHS) 2002, on the other hand, included all of mainland China, all adults and ethnic groups, and the entire economic spectrum (6,7). Therefore, the data from the CNNHS 2002 are expected to provide a comprehensive update to the results of InterASIA.

CNNHS 2002 was conducted by the Ministry of Health, the Ministry of Science and Technology, and the State Statistical Bureau to obtain a timely understanding of the changes in dietary consumption and health status. This was the first nationwide population-based comprehensive nutrition and health survey in China. It included components on diabetes, hypertension, and dyslipidemia and was conducted in 31 provinces, autonomous regions, and municipalities. We used data from CNNHS 2002 to estimate the prevalence of type 2 diabetes and impaired fasting glucose (IFG) and the association between diabetes and obesity in Chinese adults, and to compare the results with those reported from the US National Health and Nutrition Examination Survey (NHANES) 1999-2002 (8).

## Methods

The design and detailed methods of CNNHS 2002 have been described previously (6,9). Briefly, all of the 2,860 counties, districts, and cities in China were classified into 6 economic strata (large cities; medium or small cities; rural 1, 2, 3, and 4) by the government, on the basis of their administrative division, population, and level of economic development (eg, gross of products and personal income). A stratified 4-stage random cluster sampling procedure was used (6,9). A response rate of approximately 90% resulted in 272,023 participants, aged 2 to 101 years. About one-third of them were randomly selected to complete additional dietary and physical activity assessments and laboratory tests. The response rate was 91% for the additional tests. The data from 47,729 participants aged 20 years or older who completed the survey and the additional dietary, physical activity, and laboratory assessments were used in this analysis. All medical history interviews and physical examinations were conducted by trained physicians following a standardized protocol. The procedures for blood collection and processing have been described previously (7).

We defined diabetes by using the American Diabetes Association (ADA) 2009 criteria (10) (fasting plasma glucose ≥7.0 mmol/L [≥126 mg/dL]) or self-reported current diabetes treatments in the survey. We defined IFG by using ADA criteria (fasting plasma glucose of 5.6-6.9 mmol/L [100-125 mg/dL]) in people who were not diagnosed with diabetes. Body mass index (BMI) was calculated as weight in kilograms divided by height in meters squared. Overweight was defined as a BMI of 25.0 kg/m^2^ to 29.9 kg/m^2^, and obesity was defined as a BMI of at least 30.0 kg/m^2^ (11). For comparison purposes, we also used the World Health Organization (WHO) recommendation for Asian populations (12), which defined overweight as a BMI of 23.0 kg/m^2^ to 24.9 kg/m^2^ and obesity as a BMI of at least 25.0 kg/m^2^.

The following 18 cities were designated as "large cities," which are also the most economically developed cities in China: Beijing, Shanghai, Tianjin, Chongqing, Harbin, Shenyang, Dalian, Jinan, Qingdao, Ningbo, Nanjing, Zhengzhou, Shenzhen, Guangzhou, Chengdu, Xian, Wuhan, and Xiamen. Residents of rural areas of China are predominately engaged in farm work and live in villages but not in cities or towns, and residents of urban areas are not engaged in farm work and live in cities or towns. Although the living standard of the people in China has improved because of economic reforms during the past 2 decades, the improvements are uneven between urban and rural areas. Rural areas are much less developed than urban areas, especially large cities. The lifestyles of Chinese in urban areas in 2002, particularly in large cities, were also quite different from those of Chinese in rural areas. Residents of urban areas were more sedentary and more exposed to high-calorie foods than residents of rural areas (1).

China has 56 ethnic groups. Han is the largest and constitutes most of the population in China. The other 55 ethnic groups reside in 50% to 60% of the total geographic area and constitute only 6.6% of the population. Therefore, we divided CNNHS 2002 data into rural areas (rural 1, 2, 3, and 4) and urban areas (all large, medium, and small cities), or large cities only, or into Han and all minorities to explore possible differences in the prevalence of diabetes or IFG by these economic, lifestyle, and ethnic differences. The US data used for comparisons were from NHANES 1999-2002, the survey design of which was adopted by CNNHS 2002.

The protocol of the survey was approved by the Ethical Committee of the National Institute for Nutrition and Food Safety and Chinese Center for Disease Control and Prevention. Signed informed consent forms were obtained from the participants.

To provide estimates that were representative of the Chinese population, we used sampling weights. These weights account for the stratified multistage random cluster design and the unequal probabilities of selection resulting from the design (6,9). For comparison purposes, we estimated sex- and age-standardized (by direct method) prevalence rates and their 95% confidence intervals (CIs) (13). Two estimated proportions are considered significantly different at *P* < .05 if one does not fall within the 95% CI of the other. The standard population used in the direct method was the US 2000 census population or the China 2000 census population. A *t* test (13), which is equivalent to testing the difference of 2 proportions, was also used to test prevalence differences among subgroups. Logistic regression with sampling weights was used to assess association between BMI categories and prevalence of diabetes while adjusting for conventional variables. Significance was set at *P* < .05. SAS version 9.1 (SAS Institute, Inc, Cary, North Carolina) was used for all analyses.

## Results

The overall prevalence of diabetes (standardized to the China 2000 census population) was 2.7% (Table 1). Prevalence of diabetes was similar among men and women overall; this was also true by region and ethnicity. Prevalence increased with age in each regional, ethnic, and sex group. Prevalence was approximately 2 times higher overall and by age group among people who lived in urban rather than rural areas. Prevalence of diabetes was highest (6.1%) among people who lived in the 18 large cities. Among all minorities, overall and sex-specific prevalence of diabetes was significantly lower than that among Han.

The overall prevalence of IFG was 4.9% (Table 2). Prevalence among men was significantly higher than that among women; this was also true by ethnicity and in urban areas and in large cities but not in rural areas. The prevalence of IFG increased with age for each regional, ethnic, and sex group. Among people who lived in urban areas, prevalence of IFG was approximately 1.5 times higher overall and by age than that among those who lived in rural areas. Prevalence of IFG was highest (8.1%) among people who lived in large cities. Overall prevalence of IFG was significantly lower among all minorities than among Han.

The overall standardized prevalence of diabetes in China (standardized to the US 2000 census population) (3.3%) was significantly lower than that in the United States (9.3%) (8) (Table 3). The standardized prevalence of diabetes among residents of the 18 large cities was also significantly lower than that among the US population (8). However, prevalence of diabetes was similar in China and the United States among women and young (20-39 y) and middle-aged (40-59 y) adults who lived in large cities (8). The overall standardized prevalence of IFG in China (5.5%) was significantly lower than that in the United States (26%) (8); this was also true by age and sex for IFG. In the United States, IFG was consistently much more prevalent than diabetes among every age group, but in China, the differences were smaller.

**Figure 1:**
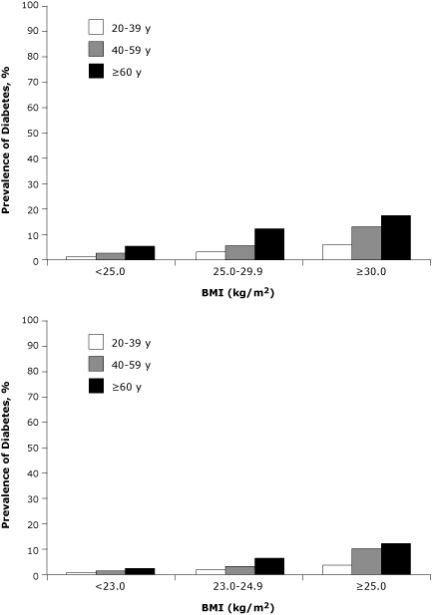
Prevalence of diabetes by age and BMI category, China National Nutrition and Health Survey, 2002. Standardized by age and sex to the 2000 China census population. All values are significantly higher at *P* < .01 compared with the same age group of the lower body mass index (BMI) categories.

**Figure 2:**
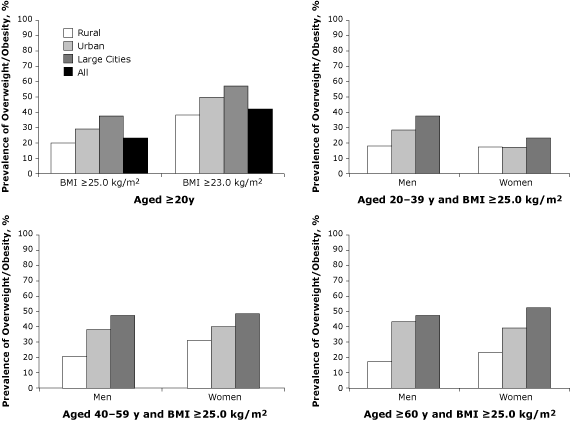
Prevalence of overweight and obesity by region, or by age, sex, and region, China National Nutrition and Health Survey, 2002. Standardized by age and sex to the 2000 China census population. Residents of urban areas are not engaged in farm work and live in large, medium, or small cities or towns. Large cities are Beijing, Shanghai, Tianjin, Chongqing, Harbin, Shenyang, Dalian, Jinan, Qingdao, Ningbo, Nanjing, Zhengzhou, Shenzhen, Guangzhou, Chengdu, Xi'an, Wuhan, and Xiamen. Residents of rural areas are predominately engaged in farm work and live in villages but not in cities or towns. All values for urban areas and large cities are significant at *P* < .01 compared with rural areas. Abbreviation: BMI, body mass index; NC, not calculated.

Obesity was strongly associated with diabetes. The overall prevalence of diabetes (standardized to the China 2000 census population) was 1.8%, 4.2%, and 7.8% in the normal, overweight, and obese groups, respectively. According to WHO's suggested BMI categories for Asians, the corresponding proportions were 1.4%, 3.3%, and 4.7%. The prevalence of diabetes increased significantly (*P* < .01) with BMI category for all age groups (Figure 1). Overweight and obesity were significantly (*P* < .01) more prevalent in urban areas than in rural areas (29.4% vs 20.1% on the basis of the general BMI categories, or 49.1% vs 38.0% on the basis of WHO's suggested BMI categories for Asians) (Figure 2). The 18 large cities had the highest prevalence among all areas regardless of which BMI category was used; this was also true by age group (Figure 2). Approximately half of women and men aged 40 years or older were overweight or obese (BMI ≥25 kg/m^2^). Prevalence of diabetes was significantly higher among obese and overweight participants than among normal-weight participants (odds ratio [OR], 4.12; 95% CI, 4.12-4.13, and OR, 2.18; 95% CI, 2.18-2.18, respectively) after adjusting for age, sex, region, ethnicity, education, and smoking (data not shown).

**Table:** 

**BMI Category, kg/m^2^**	**20-39 y, %**	**40-59 y, %**	**≥60 y, %**
<25.0	0.6	2.0	4.9
25.0-29.9	2.3	5.4	11.6
≥30.0	5.4	12.3	16.9
<23.0	0.5	1.0	2.3
23.0-24.9	1.8	3.4	6.3
≥25.0	3.7	10.8	13.0

**Table:** 

**Characteristic**	**Region**			

	**Rural**	**Urban**	**Large Cities**	**All**
**BMI, %**				
**≥**25 kg/m^2^	20.1	29.4	37.4	23.2
**≥**23 kg/m^2^	38.0	49.1	56.9	41.8
**Age/sex, %**				
**20-39 y**				
Men	18.1	28.8	37.5	NC
Women	17.4	17.5	22.7	NC
**40-59 y**				
Men	20.9	39.1	47.9	NC
Women	31.4	40.6	49.4	NC
**≥60 y**				
Men	17.5	44.6	48.2	NC
Women	23.7	40.3	53.9	NC

## Discussion

Our findings of higher prevalence of overweight and obesity, diabetes, and IFG in urban areas, especially large cities, compared with rural areas, may be explained by the improved standard of living in China during the past 2 decades. These improvements also led to considerable reduction in daily physical activity and to an increase in consumption of high-calorie foods, especially in urban areas (1,14). In 2002, urban residents consumed an average of 35.4% of their energy from fat; the upper limit suggested by WHO is 30% (1).

Our findings that the prevalence of diabetes was higher among urban than rural residents and was similar among men and women but that the prevalence of IFG was significantly higher among men than women were also observed in InterASIA (3). However, contrary to our finding, InterASIA reported that urban and rural residents had similar prevalence of IFG (3). Among participants aged 35 to 74 years who lived in urban areas, the overall and sex-specific prevalence of diabetes from the present study were significantly lower than those reported from InterASIA (3) (6.0% [95% CI, 5.5%-6.6%] vs 7.8%); the same finding was true for rural areas (2.6% [95% CI, 2.3%-2.9%] vs 5.1%). These differences may be because CNNHS 2002 covered more cities and underdeveloped rural areas than did InterASIA (3,5). Our results, which are based on more representative national data, update those reported from InterASIA.

Contrary to findings among US minorities, such as American Indians (15), the prevalence of diabetes and of IFG among all minorities in China was significantly lower than that among Han. This difference may be because more than 80% of minorities in China reside in rural areas. Economic developments between urban and rural areas have been uneven, and residents of rural areas are still predominately engaged in labor-intensive farm work or labor-intensive temporary work in cities.

The finding that the prevalence of diabetes among young and middle-aged adults who lived in large cities was similar to the prevalence among those age groups in the United States (8) was consistent with the rapidly rising prevalence of overweight and obesity in China, especially among young and middle-aged adults (1). The prevalence of overweight and obesity (BMI ≥25.0 kg/m^2^) among adults aged 18 years or older increased by 49.3%, from 14.6% in 1992 to 21.8% in 2002, and the prevalence among adults aged 18 to 44 years almost tripled (1). The data from CNNHS 2002 showed an even higher prevalence of overweight and obesity among adults aged 20 years or older, particularly those who lived in large cities. Overweight and obesity are typically associated with sustained peripheral and hepatic insulin resistance, which may eventually lead to diabetes (16). IFG was consistently much more prevalent than diabetes in each age group in the United States, but the reasons for the smaller differences in China are unclear and need further study.

Our study has several limitations. First, the CNNHS 2002 provided only cross-sectional data and did not permit further study of risk factors for incident diabetes or IFG. Second, related social data about people's knowledge, attitudes, and beliefs about the factors contributing to health and disease were unavailable. This limited our ability to further explore why diabetes and IFG were less prevalent among all minorities than among Han or were less prevalent in rural areas than in urban areas, although in general residents of rural areas in China had lower living standards, lower income, and fewer benefits such as retirement and health care than residents of urban areas. This pattern of lower socioeconomic status being associated with lower prevalence of diabetes and of IFG is the reverse of the disease patterns usually observed in the United States. Third, the comparisons in this study were done only for diabetes defined by the ADA fasting plasma glucose criteria because oral glucose tolerance tests were not performed in NHANES 1999-2002 (8). However, from CNNHS 2002 data, the prevalence of diabetes found by including the additional diabetes cases detected by oral glucose tolerance testing of participants with fasting plasma glucose of 5.6 mmol/L to 6.9 mmol/L was similar to, though slightly higher than the prevalence based on the ADA criteria in China (data not shown).

Our observations suggest that, in view of China's rapid urbanization, the prevalence of diabetes there may increase to a level similar to that of the United States soon. Adults who live in urban areas of China, especially in large cities, should modify their lifestyles to reduce weight and to minimize the future social and economic burden of diabetes.

## Figures and Tables

**Table 1:** Prevalence of Type 2 Diabetes^a^ by Age, Sex, Region, and Ethnicity, China National Nutrition and Health Survey, 2002

Characteristic	**% (95% CI)^b^**			

	**20-39 y, n = 17,978**	**40-59 y, n= 20,665**	**≥60 y, n = 9,086**	**≥20 y^c^, n = 47,729**
**All**	1.0 (0.8-1.2)	3.5 (3.2-3.9)	7.6 (6.8-8.4)	2.7 (2.5-2.8)
Men	0.9 (0.6-1.2)	3.3 (2.8-3.8)	7.4 (6.3-8.4)	2.5 (2.3-2.8)
Women	1.0 (0.7-1.2)	3.7 (3.2-4.2)	7.9 (6.7-9.0)	2.8 (2.5-3.0)
**Region^d^**				
**Urban**	1.1 (0.7-1.5)	6.1 (5.3-7.0)	12.8 (11.2-14.3)	3.9 (3.6-4.3)
Men	1.0 (0.4-1.5)	6.5 (5.3-7.8)	12.7 (10.5-14.9)	3.9 (3.4-4.5)
Women	1.3 (0.7-1.8)	5.8 (4.8-6.9)	12.9 (10.7-15.1)	3.9 (3.4-4.5)
**Large cities**	2.0 (1.3-2.6)	9.5 (8.4-10.6)	16.9 (15.2-18.7)	6.1 (5.6-6.7)
Men	2.2 (1.2-3.3)	9.5 (7.9-11.1)	15.5 (13.1-17.9)	5.9 (5.1-6.7)
Women	1.8 (0.9-2.6)	9.5 (8.1-10.9)	18.3 (15.8-20.8)	6.4 (5.7-7.2)
**Rural**	0.9 (0.7-1.1)	2.4 (2.0-2.7)	4.3 (3.5-5.0)	1.9 (1.6-2.1)
Men	0.9 (0.6-1.3)	2.0 (1.5-2.5)	3.9 (2.8-4.9)	1.7 (1.4-2.0)
Women	0.8 (0.5-1.1)	2.7 (2.2-3.2)	4.7 (3.6-5.8)	2.0 (1.7-2.3)
**Ethnicity^e^**				
**Han**	1.0 (0.8-1.2), n = 16,042	3.6 (3.3-4.0), n = 18,716	7.9 (7.1-8.8), n = 8,260	2.8 (2.6-3.0), n = 43,018
Men	0.9 (0.6-1.2)	3.4 (2.9-4.0)	7.6 (6.5-8.8)	2.6 (2.3-2.9)
Women	1.0 (0.7-1.3)	3.8 (3.3-4.3)	8.3 (7.1-9.5)	2.9 (2.6-3.2)
**Minorities**	0.5 (0.1-0.9), n = 1,936	1.9 (1.1-2.6), n = 1,949	3.3 (1.8-4.8), n = 826	1.3 (0.9-1.7), n = 4,711
Men	0.8 (0.1-1.6)	1.4 (0.5-2.4)	3.5 (1.3-5.6)	1.4 (0.8-2.0)
Women	0.2 (0.1-0.6)	2.2 (1.1-3.3)	3.1 (1.1-5.2)	1.2 (0.7-1.7)

Abbreviation: CI, confidence interval.

a Defined as fasting plasma glucose of at least 7.0 mmol/L (≥126 mg/dL) or self-reported current diabetes treatments.

b Two estimated proportions are considered significantly different at *P* < .05 if one does not fall within the 95% CI of the other.

c Standardized by sex and age to the 2000 China census population.

d Residents of urban areas are not engaged in farm work and live in large, medium, or small cities or towns. Large cities are Beijing, Shanghai, Tianjin, Chongqing, Harbin, Shenyang, Dalian, Jinan, Qingdao, Ningbo, Nanjing, Zhengzhou, Shenzhen, Guangzhou, Chengdu, Xi'an, Wuhan, and Xiamen. Residents of rural areas are predominately engaged in farm work and live in villages but not in cities or towns.

e Han ethnicity in China constitutes 93.4% of the population. Minorities defined as all 55 minority ethnicities in China, which constitute 6.6% of the population.

**Table 2:** Prevalence of Impaired Fasting Glucose^a^ by Age, Sex, Region, and Ethnicity, China National Nutrition and Health Survey, 2002

Characteristic	**% (95% CI)^b^**			

	**20-39 y, n = 17,978**	**40-59 y, n= 20,665**	**≥60 y, n = 9,086**	**≥20 y^c^, n = 47,729**
**All**	3.2 (2.8-3.6)	6.2 (5.7-6.6)	9.2 (8.4-10.1)	4.9 (4.6-5.1)
Men	3.8 (3.2-4.4)	6.6 (5.9-7.3)	8.8 (7.7-10.0)	5.3 (4.8-5.7)
Women	2.7 (2.2-3.1)	5.8 (5.2-6.4)	9.7 (8.4-10.9)	4.6 (4.2-5.0)
**Region^d^**				
**Urban**	4.3 (3.5-5.1)	7.7 (6.8-8.6)	12.5 (10.9-14.0)	6.1 (5.6-6.7)
Men	5.6 (4.3-6.9)	9.0 (7.6-10.5)	11.9 (9.8-14.1)	7.1 (6.2-8.0)
Women	3.3 (2.3-4.2)	6.6 (5.5-7.8)	13.0 (10.9-15.2)	5.4 (4.7-6.0)
**Large cities**	6.2 (5.0-7.3)	9.4 (8.4-10.5)	14.5 (12.9-16.1)	8.1 (7.4-8.8)
Men	9.2 (7.1-11.2)	11.8 (10.3-13.6)	15.3 (12.9-17.7)	10.6 (9.4-11.9)
Women	3.7 (2.5-4.9)	7.6 (6.3-8.8)	13.8 (11.6-16.0)	6.1 (5.4-6.9)
**Rural**	2.8 (2.4-3.2)	5.5 (5.0-6.0)	7.2 (6.2-8.1)	4.2 (3.9-4.5)
Men	3.1 (2.5-3.7)	5.6 (4.8-6.4)	6.8 (5.5-8.2)	4.4 (3.9-4.8)
Women	2.5 (2.0-3.0)	5.4 (4.6-6.1)	7.5 (6.1-8.9)	4.2 (3.7-4.6)
**Ethnicity^e^**				
**Han**	3.3 (2.9-3.6), n = 16,042	6.3 (5.8-6.7), n = 18,716	9.6 (8.7-10.5), n = 8,260	5.0 (4.7-5.3), n = 43,018
Men	3.8 (3.2-4.4)	6.6 (5.9-7.3)	9.0 (7.8-10.3)	5.3 (4.9-5.7)
Women	2.8 (2.2-3.2)	6.0 (5.3-6.6)	10.1 (8.8-11.5)	4.8 (4.4-5.2)
**Minorities**	2.5 (1.6-3.4), n = 1,936	4.8 (3.7-6.0), n = 1,949	5.1 (3.3-7.0), n = 826	3.4 (2.8-4.0), n = 4,711
Men	3.3 (1.9-4.8)	6.7 (4.7-8.7)	6.2 (3.4-9.0)	4.4 (3.4-5.5)
Women	1.9 (0.8-2.9)	3.5 (2.1-4.8)	4.2 (1.9-6.6)	2.6 (1.8-3.3)

Abbreviation: CI, confidence interval.

a Defined as fasting plasma glucose of 5.6 mmol/L to 6.9 mmol/L (100-125 mg/dL) in people who were not diagnosed with diabetes.

b Two estimated proportions are considered significantly different at *P* < .05 if one does not fall within the 95% CI of the other.

c Standardized by sex and age to the 2000 China census population.

d Residents of urban areas are not engaged in farm work and live in large, medium, or small cities or towns. Large cities are Beijing, Shanghai, Tianjin, Chongqing, Harbin, Shenyang, Dalian, Jinan, Qingdao, Ningbo, Nanjing, Zhengzhou, Shenzhen, Guangzhou, Chengdu, Xi'an, Wuhan, and Xiamen. Residents of rural areas are predominately engaged in farm work and live in villages but not in cities or towns.

e Han ethnicity in China constitutes 93.4% of the population. Minorities defined as all 55 minority ethnicities in China, which constitute 6.6% of the population.

**Table 3:** Prevalence of Type 2 Diabetes and Impaired Fasting Glucose by Region, China National Nutrition and Health Survey, 2002, and the US National Health and Nutrition Examination Survey, 1999-2002

Region	**% (95% CI)^a^**					

	20–39 y	40–59 y	≥60 y	**≥20 y^b^**		

				Total	**Men**	**Women**
**Diabetes^c^**						
**China** **d**	0.9 (0.7-1.1)	3.4 (3.0-3.7)	7.3 (6.3-8.2)	3.3 (3.0-3.5)	3.0 (2.7-3.4)	3.4 (3.0-3.8)
Urban	1.0 (0.6-1.4)	5.9 (5.1-6.7)	12.0 (10.1-14.0)	5.3 (4.8-5.8)	5.2 (4.5-5.9)	5.3 (4.5-6.1)
Large cities	1.9 (1.3-2.6)	8.9 (7.9-9.9)	16.5 (14.6-18.5)	7.8 (7.2-8.4)	7.3 (6.4-8.2)	8.3 (7.3-9.2)
Rural	0.8 (0.6-1.1)	2.3 (1.9-2.6)	4.2 (3.3-5.1)	2.1 (1.9-2.4)	1.9 (1.6-2.2)	2.4 (1.9-2.8)
**United States^e^**	2.4	9.8	21.1	9.3	10.6	8.2
**Impaired fasting glucose^f^**						
**China** **d**	2.9 (2.6-3.3)	6.1 (5.6-6.5)	9.1 (8.0-10.2)	5.5 (5.2-5.8)	5.7 (5.2-6.1)	5.5 (5.0-5.9)
Urban	3.9 (3.2-4.6)	7.7 (6.7-8.6)	12.3 (10.3-14.3)	7.2 (6.6-7.8)	7.9 (6.9-8.8)	6.8 (5.9-7.7)
Large cities	5.9 (4.8-7.0)	9.5 (8.4-10.6)	14.4 (12.6-16.3)	9.2 (8.4-9.9)	11.4 (10.2-12.6)	7.4 (6.4-8.3)
Rural	2.5 (2.1-2.9)	5.4 (4.9-5.9)	7.0 (5.8-8.2)	4.6 (4.2-5.0)	4.6 (4.1-5.1)	4.7 (4.1-5.3)
**United States^e^**	15.7	29.8	37.9	26.0	32.8	19.5

Abbreviation: CI, confidence interval.

a Two estimated proportions are considered significantly different at *P* < .05 if one does not fall within the 95% CI of the other.

b Standardized to the 2000 US census population.

c Defined as fasting plasma glucose of at least 7.0 mmol/L (≥126 mg/dL) or self-reported current diabetes treatments.

d Residents of urban areas are not engaged in farm work and live in large, medium, or small cities or towns. Large cities are Beijing, Shanghai, Tianjin, Chongqing, Harbin, Shenyang, Dalian, Jinan, Qingdao, Ningbo, Nanjing, Zhengzhou, Shenzhen, Guangzhou, Chengdu, Xi'an, Wuhan, and Xiamen. Residents of rural areas are predominately engaged in farm work and live in villages but not in cities or towns.

e Source: Cowie et al (8).

f Defined as fasting plasma glucose of 5.6 mmol/L to 6.9 mmol/L (100-125 mg/dL) in people who were not diagnosed with diabetes.
